# Curcumin-mediated antimicrobial photodynamic therapy prevents *Candida* biofilm formation in a species-dependent manner

**DOI:** 10.1007/s10103-026-04924-2

**Published:** 2026-06-20

**Authors:** João Salviano Simões Chagas da Silva, Lorena Cristina da Mata, Valdirene Neves Monteiro, Evandro Leão Ribeiro, Lucas Danilo Dias, Plínio Lázaro Faleiro Naves

**Affiliations:** 1 Laboratório de Bioensaios, Universidade Estadual de Goiás, Anápolis, Brazil; 2 Instituto de Patologia Tropical e Saúde Pública, Universidade Federal de Goiás, Goiânia, Brazil; 3 Laboratório de Novos Materiais, Universidade Evangélica de Goiás, Anápolis, Brazil

**Keywords:** Antimicrobial photodynamic therapy, Stage-dependent antibiofilm activity, Curcumin, Candida biofilms

## Abstract

This study aimed to systematically evaluate the antibiofilm efficacy of curcumin-mediated antimicrobial photodynamic therapy (aPDT) activated by 450 nm LED light against biofilm formation and mature biofilms of clinically relevant *Candida* species, with particular emphasis on preventive potential and species-dependent susceptibility; Biofilm formation and maturation were investigated in five standard *Candida* strains. Biofilm formation (preventive approach) and established biofilms were treated with curcumin followed by irradiation with 450 nm LED light. Biofilm biomass was quantitatively assessed using the crystal violet assay. Comparative analyses were performed to determine species-dependent responses to aPDT. Data normality was assessed using the Shapiro-Wilk test, and intergroup comparisons were performed using the Kruskal-Wallis test followed by Dunn’s post hoc test when appropriate. All *Candida* strains demonstrated a pronounced ability to form biofilms, being classified as strong biofilm producers, except *Candida dubliniensis*, which exhibited moderate biofilm formation. Curcumin-mediated aPDT significantly inhibited biofilm development across all tested species (*p* < 0.05), achieving reductions of 86.82% for *C. metapsilosis*, 85.05% for *C. orthopsilosis*, 83.33% for *C. parapsilosis*, 75.34% for *C. dubliniensis*, and 68.19% for *C. albicans*. This preventive antibiofilm effect is likely associated with reactive oxygen species generation, resulting in oxidative damage to essential cellular structures and impairment of early extracellular matrix establishment. In contrast, aPDT activity against mature biofilms was significantly attenuated and highly species-dependent, with reductions ranging from 49.63% (*C. parapsilosis*) to 0.64% (*C. dubliniensis*); however, these reductions were not statistically significant (*p* > 0.05). Curcumin-mediated aPDT exerted a significant preventive antibiofilm effect against *Candida* biofilm formation but showed limited and non-significant activity against mature biofilms. These findings position curcumin-mediated aPDT as a promising strategy for preventing *Candida* biofilm establishment, while indicating that improved photosensitizer delivery and optimized irradiation protocols are required to enhance activity against established biofilms.

##  Introduction

Biofilms are complex, three-dimensional microbial communities comprising single or multiple species. They can attach to biological surfaces, like host tissues, or abiotic surfaces, such as medical devices. These communities are enveloped in an extracellular polymeric substance (EPS) matrix, primarily composed of polysaccharides, proteins, and nucleic acids. This matrix acts as a protective barrier, making biofilms resistant to antimicrobial agents and host immune responses [21].

Infections caused by human pathogenic fungi, particularly *Candida*, represent a significant medical challenge due to their ability to disseminate throughout the body. *Candida* infections associated with biofilms are especially difficult to treat as they are recalcitrant and exhibit resistance to antifungals. The limited availability of therapeutic classes and antifungals further complicates treatment [16].

Research on *C. albicans* biofilms indicates that preventing their initial formation is more effective than treating mature, more resistant biofilms [5]. One alternative strategy to inhibit *C. albicans* biofilm formation involves modifying the chemical properties of biomaterial surfaces to prevent or reduce biofilm development [13]. Alternative therapies are being explored to combat fungal biofilm infections. These include phytoextracts and nanoparticles, which can act as carriers for EPS matrix disrupters [1].

Antimicrobial photodynamic chemotherapy (aPDT) is another promising approach, involving the use of a light source, molecular oxygen (O_2_) and a photosensitizer (PS) to generate reactive oxygen species (ROS), leading to cell death. This therapy is being investigated against various microbial biofilms [13]. Photosensitizers, such as tetrapyrroles, synthetic dyes, and natural compounds, are activated by specific light wavelengths in the presence of oxygen to produce ROS. This ROS production induces oxidative stress and cytotoxicity within the cell, resulting in microorganism death [31].

Among the PS applied, curcumin is able to generate ROS that cause severe and irreversible oxidative stress in microbial cells. This multifaceted attack targets various cellular components simultaneously. For instance, the plasma membrane undergoes lipid peroxidation, leading to structural integrity loss, increased permeability, and the leakage of intracellular contents. ROS also oxidizes and denatures essential proteins and enzymes. Furthermore, oxidative damage to nucleic acids (DNA and RNA) can cause strand breaks and mutations, rendering genetic replication and transcription inviable [12]. The broad range of mechanisms and low propensity for resistance selection associated with photodynamic inactivation of microorganisms lead to a combination of cellular structure and function damage, resulting in rapid death of microorganisms, including Gram-positive and Gram-negative bacteria, fungi, and enveloped viruses [9, 10, 24].

Given the increasing resistance of yeasts to conventional antifungals and the challenges in managing *Candida* biofilm-associated infections [13, 25], this study aimed to evaluate the efficacy of curcumin-mediated aPDT using 450 nm LED light in both inhibiting biofilm formation (preventive approach) and reducing pre-formed mature biofilms of five standard *Candida* strains.

##  Methodology

###  Microorganisms evaluated

Five standard strains of *Candida* yeasts were evaluated: *C. albicans* ATCC 10,231, *C. metapsilosis* ATCC 96,143, *C. dubliniensis* ATCC MYA-646, *C. orthopsilosis* ATCC 96,141, and *C. parapsilosis* ATCC 22,019.

###  Curcumin preparation

The curcumin stock solution was obtained by weighing 16.2 mg of synthetic curcumin (98%) (PDT Pharma, Brazil) and dissolving it in 20 mL of 96° ethanol (Itajá, Jalles Machado, Brazil). For complete dissolution, this solution was placed in an ultrasonic bath at 40 kHz (Ultronique Q3.8/40A, Indaiatuba, São Paulo) for 3 min. From the stock solution, aqueous solution of 320 µM were prepared for use in the assays.

###  Biofilm formation

Biofilm formation was determined by assessing the total biomass of yeasts adhered to the wells using the crystal violet method [33]. The *Candida* strains were stored under standard laboratory conditions (cryopreserved stocks at − 80 °C) and routinely maintained on Sabouraud agar prior to use. Yeasts were reactivated on Sabouraud agar (FIRSTLAB, MicroMedia, Hungary) incubated at 37 °C for 72 h. Subsequently, typical colonies were resuspended in 5 mL of sterile physiological saline (SPS), and the inoculum density was adjusted using the 0.5 McFarland scale. Then, 500 µL of the adjusted inocula were transferred to tubes containing 4,500 µL of Brain Heart Infusion broth with 2% sucrose (BHIS) (HIMEDIA^®^, HiMedia Laboratories, India). After homogenization of the tubes, 100 µL of the broths with the inocula were transferred to wells of 96-well flat-bottom polystyrene microplates (Cralplast, Cral, Brazil), which were incubated at 37 °C for 48 h for biofilm formation.

After incubation, the microplates were processed by removing the broth with total growth, washing the wells, fixing the adhered biofilms with 96% ethanol (Itajá, Jalles Machado, Brazil), staining with 0.1% crystal violet (Newprov, Brazil), removing excess dye with three washes with distilled water in an automatic microplate washer Aquari^®^ (MA 615, Brazil), and drying at room temperature (RT). Subsequently, 200 µL of 33% glacial acetic acid were added to the wells, and after incubation at RT for 20 min.

Optical densities (OD_620nm_) were then read in a microplate spectrophotometer (MultiskanFC, ThermoScientific, China). Based on the OD_620nm_ values obtained from non-inoculated controls (NIC) and adhered yeasts (AY), biofilm formation was classified as weak (OD_AY_ < 3xOD_NIC_), moderate (3xOD_NIC_ < OD_AY_ < 6xOD_NIC_), or strong (OD_AY_ > 6xOD_NIC_) [33].

###  Effect of photodynamic therapy on biofilm formation

The effect of curcumin-mediated photodynamic therapy on biofilm formation (preventive approach**)** was evaluated as described for the biofilm formation study. Briefly, the microplate was prepared as previously mentioned with the addition of 100 µL of the adjusted inocula to wells in columns 2 to 11. In wells of columns 2 to 6, an additional 100 µL of the curcumin solution was added to achieve a final concentration of 320 µM. In the wells of the remaining columns, only the inoculum containing curcumin was added and maintained in the absence of light, serving as the non-photoactivated control (NPC). Yeasts were added to the microplate rows as follows: row B for *C. albicans* ATCC 10,231, row C for *C. metapsilosis* ATCC 96,143, row D for *C. dubliniensis* ATCC MYA-646, row E for *C. orthopsilosis* ATCC 96,141, row F for *C. parapsilosis* ATCC 22,019, and row G for the Non-Inoculated Control (NIC).

**Fig. 1:**
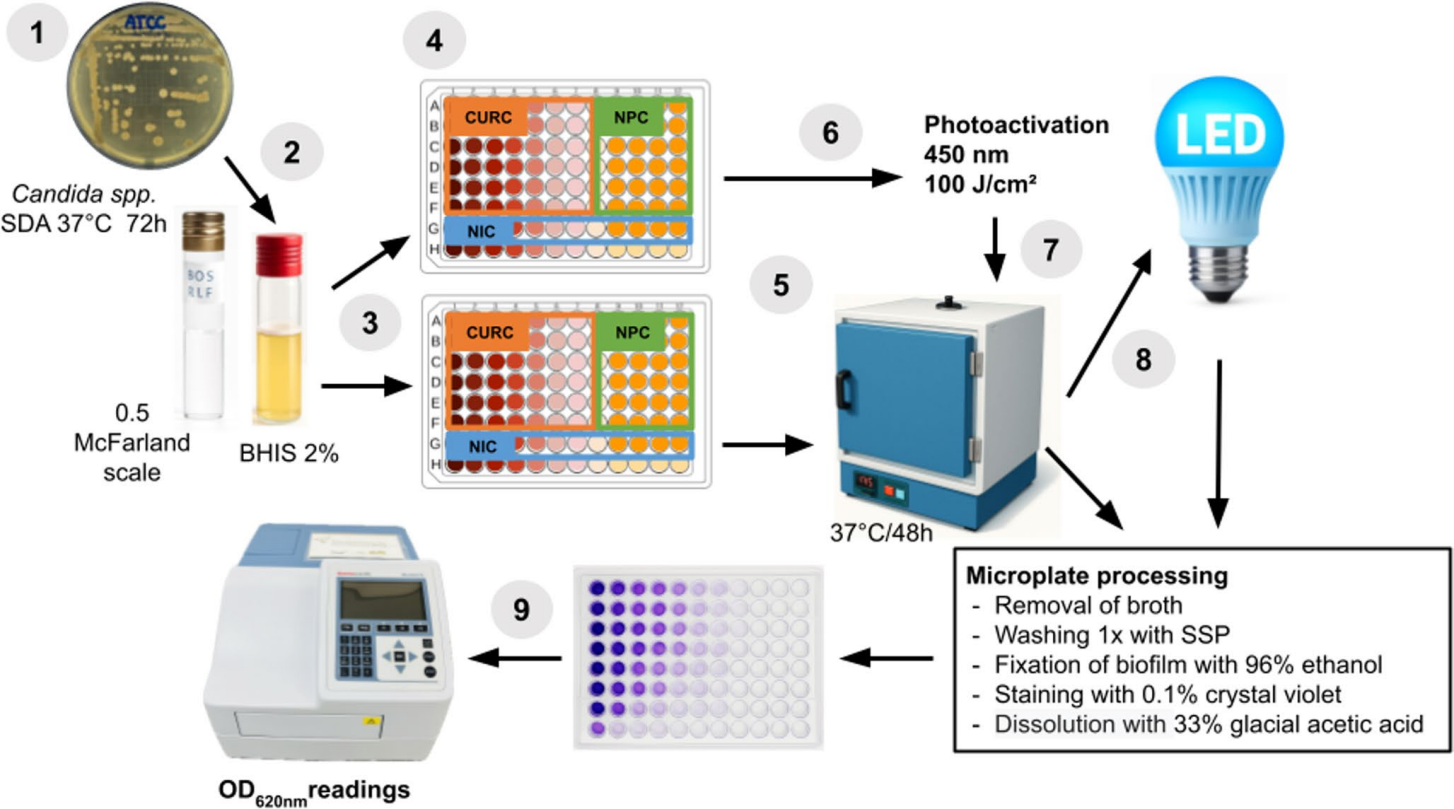
Evaluation of photodynamic therapy on biofilm formation and against pre-formed *Candida* spp. biofilms. 1. Incubation of Candida spp. on Sabouraud agar at 37°C for 72 hours 2. Adjustment of the inoculum density in Brain Heart Infusion broth with 2% sucrose (BHIS) to a 0.5 McFarland scale 3. Transfer of 100 μL of the adjusted inocula to microplate wells 4. Addition of 100 μL of 640 μM curcumin to the microplate wells 5. Incubation at RT for 20 minutes in the dark (pre-irradiation period) 6. Photoactivation with 450 nm LED light prior to biofilm formation 7. Incubation of the microplates at 37°C for 48 hours 8. Photoactivation with 450 nm LED light after biofilm formation 9. Measurement of OD_620nm_ and calculation of biofilm formation. *OD*_*620nm*_ Optical density at 620 nm, *CURC* curcumin, *NPC* non-photoactivated control, *NIC* non-inoculated control

Subsequently, the microplate was incubated in the absence of light at RT for 20 min (pre-irradiation time). Then, the microplate was illuminated for 1 h and 17 min (dose of 100 J/cm^2^) using a 450 nm LED light source (0.02165 W/cm^2^) to evaluate the effect of curcumin photoactivation on biofilm formation (prior to biomass establishment). Subsequently, the microplate was incubated at 37 °C for 48 h (Fig. 1). After the incubation period, the microplate was processed as previously described.

###  Effect of photodynamic therapy on mature biofilm

The activity of photodynamic therapy on mature biofilm was evaluated by exposing pre-formed biofilms under the same conditions described in item 2.3. Microplates were prepared as previously mentioned for biofilm formation assays with incubation at 37 °C for 48 h. After mature biofilms were formed, the microplates were treated under aseptic conditions by removing the spent media and adding 100 µL of the 320 µM curcumin solution to the wells, as described in item 2.4.

Subsequently, the microplates were incubated for 20 min (pre-irradiation time) at RT in the dark. After the pre-irradiation period, the microplate was illuminated for 1 h and 17 min (dose of 100 J/cm^2^) using a 450 nm LED light source (0.02165 W/cm^2^) to evaluate the impact of curcumin-mediated photoactivation against mature (48 h) biofilm (Fig. 1). After the incubation period, the microplate was processed as previously described in item 2.3.

All experiments were performed in independent triplicates, and the results are presented as mean ± standard deviation. The OD_620_ nm values obtained from photoactivated wells containing curcumin were compared to those from non-photoactivated control wells (curcumin without light exposure).

###  Statistical analysis

Data distribution was assessed using the Shapiro-Wilk normality test. For the biofilm formation assay, normality was not observed (*p* = 0.007), and data were therefore analyzed using the Kruskal-Wallis test followed by Dunn’s post hoc test for multiple comparisons. In this essay, curcumin-treated groups were compared with their respective untreated controls, with statistical significance defined as *p* < 0.05. For the mature biofilm assay, the Shapiro–Wilk test did not reject normality *(p* = 0.284). Nevertheless, considering the small sample size and maintaining a conservative analytical framework across assays, non-parametric analysis was retained. Comparisons were performed using the Kruskal-Wallis test. No statistically significant differences were detected between curcumin-treated mature biofilms and their respective untreated controls. Statistical analyses were performed using Jamovi and BioEstat. Data are presented as mean ± standard deviation.

##  Results and discussion

###  Determination of total biomass by crystal violet method

**Fig. 2:**
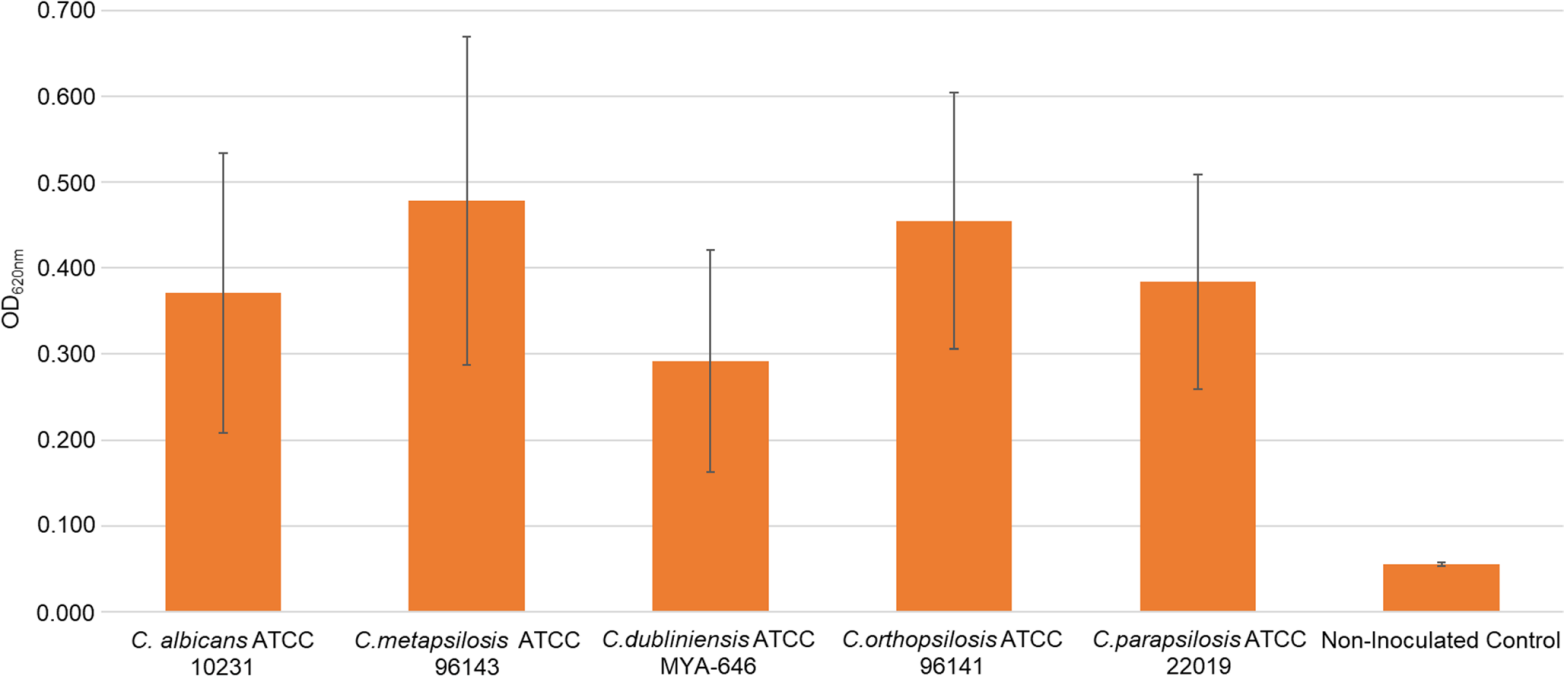
Total biomass (OD_620nm_) of *Candida* biofilms. *OD*_*620nm*_ Optical density at 620 nm, *ATCC* American type culture collection

Biofilm-forming capacity was evaluated by quantifying total adherent biomass using the crystal violet assay, which enabled the classification of each *Candida* strain according to its biofilm production profile, as shown in Fig. 2.

All yeasts evaluated formed biofilm; 4 were classified as strong biofilm producers. *C. metapsilosis* ATCC 96,143 showed the highest OD_620nm_ (0.478 ± 0.191), while *C. dubliniensis* ATCC MYA-646 presented the lowest OD_620nm_ (0.292 ± 0.129) and was the only yeast classified as a moderate biofilm producer. The other yeasts were classified as strong producers: *C. albicans* ATCC 10,231 (0.371 ± 0.163), *C. orthopsilosis* ATCC 96,141 (0.455 ± 0.149), and *C. parapsilosis* ATCC 22,019 (0.384 ± 0.125).

Previous studies have reported different biofilm formation values for *C. albicans*, depending on the origin and evaluation method employed. One study reported OD_590nm_ between 2.410 and 2.530 for *C. albicans* ATCC 10,231 biofilms [20], with results differing from those obtained in our study (OD_620nm_ = 0.371 ± 0.163). This divergence should not be interpreted as biological inconsistency alone, since crystal violet quantification is highly sensitive to methodological variables, including inoculum density, incubation time, washing stringency, dye retention, destaining solvent, and optical wavelength.

Another study analyzed clinical samples of *C. albicans* and *C. dubliniensis* isolated from sputum of cystic fibrosis patients and recorded OD_570nm_ between 0.280 and 0.640 for *C. albicans* and a higher value for *C. dubliniensis* (OD_570nm_ = 1.780) [15]. Although our results for *C. albicans* fall within this range, it is important to note that direct comparison is limited due to differences in isolates and methodology used in each experiment. Furthermore, our study used ATCC standard strains, unlike the other work.

*C. metapsilosis* ATCC 96,143, *C. orthopsilosis* ATCC 96,141, and *C. parapsilosis* ATCC 22,019 were previously classified as having “weak biofilm formation” [28]. In another previous study, the biofilm of different *Candida* species, including strains of *C. albicans*, *C. parapsilosis*, *C. orthopsilosis*, *C. metapsilosis*, and *C. parapsilosis* ATCC 22,019, was analyzed, and the microorganisms showed OD_595nm_ = 1.761 ± 0.412 [18]. Together, these discrepancies reinforce that biofilm classification is not an intrinsic and fixed property of the strain, but rather an experimentally dependent phenotype strongly influenced by assay conditions.

*C. dubliniensis* ATCC MYA-646 exhibited biofilm formation with OD_620nm_ = 0.542 ± 0.108 [2]. In our study, the same strain showed OD_620nm_ = 0.292 ± 0.129 and was classified as moderate. These variations reinforce the need for standardization of protocols for fungal biofilm quantification to ensure greater reproducibility among studies.

Fungal biofilms exhibit intrinsic resistance to conventional antifungal treatments and host immune responses, making biofilm-related infections a major clinical challenge [13]. Additionally, antifungal resistance is complex and can be caused by several factors, including a response to a compound or an irreversible genetic alteration resulting from prolonged exposure to an antimicrobial [22]. Therefore, the confirmation that all tested strains formed measurable biofilms provides the experimental basis for evaluating curcumin-mediated antimicrobial photodynamic therapy against both early biofilm establishment and mature biofilm architecture.

###  Effect of photodynamic therapy on biofilm formation

Curcumin-mediated aPDT inhibited biofilm formation in all analyzed yeasts. The best inhibition results were observed for *C. metapsilosis* ATCC 96,143 (86.82%) and *C. parapsilosis* ATCC 22,019 (83.33%) compared to control groups. The lowest reduction in biofilm formation was observed in *C. albicans* ATCC 10,231 (68.19%), while *C. dubliniensis* ATCC MYA-646 and *C. orthopsilosis* ATCC 96,141 were inhibited by 75.34% and 85.05%, respectively (Table 1).

**Table 1:** OD_620nm_ of biofilms formed after curcumin-mediated photodynamic therapy and non-photoactivated controls

Candida	Curcumin (320 µM)		Non- photoactivated controls		Reductions
	Mean	SD	Mean	SD	%
*C. albicans* ATCC 10,231	**0.118**	± 0.029	**0.371**	± 0.163	68.19
*C. metapsilosis* ATCC 96,143	**0.063**	± 0.009	**0.478**	± 0.191	86.82
*C. dubliniensis* ATCC MYA-646	**0.072**	± 0.013	**0.292**	± 0.129	75.34
*C. orthopsilosis* ATCC 96,141	**0.068**	± 0.018	**0.455**	± 0.149	85.05
*C. parapsilosis* ATCC 22,019	**0.064**	± 0.009	**0.384**	± 0.125	83.33
Non inoculated control	**0.056**	± 0.004	**0.055**	± 0.002	-

*SD* Standard Deviation, *ATCC* American Type Culture Collection

Because the biofilm formation data did not meet the assumption of normality according to the Shapiro–Wilk test (*p* = 0.007), intergroup comparisons were performed using the Kruskal–Wallis test followed by Dunn’s multiple-comparison post hoc test. This analysis showed statistically significant reductions in biomass for all curcumin-mediated aPDT-treated groups compared with their respective non-photoactivated controls (*p* < 0.05), indicating that curcumin-mediated aPDT effectively interfered with the early stages of biofilm establishment across all tested *Candida* species. Importantly, the treated groups displayed OD_620nm_ values close to the non-inoculated control, particularly for *C. metapsilosis*, *C. orthopsilosis*, and *C. parapsilosis*, suggesting substantial impairment of initial adhesion and/or early biomass accumulation. Therefore, the preventive antibiofilm effect of curcumin-mediated aPDT was not only biologically evident but also quantitatively supported across the evaluated species.

The light-only control showed that blue LED irradiation alone did not inhibit *Candida* biofilm formation under the tested conditions. In the absence of curcumin, all irradiated control groups maintained substantial biofilm biomass, with OD_620nm_ values of 0.371 ± 0.163 for *C. albicans*, 0.478 ± 0.191 for *C. metapsilosis*, 0.292 ± 0.129 for *C. dubliniensis*, 0.455 ± 0.149 for *C. orthopsilosis*, and 0.384 ± 0.125 for *C. parapsilosis*. These results confirm that the preventive antibiofilm effect was driven by curcumin photoactivation rather than by blue light alone.

Previously, complete inactivation of planktonic yeast viability by curcumin-mediated aPDT (20 µM) was observed after 1 min of pre-irradiation against *Candida dubliniensis* CBS 7987 and *Candida albicans* ATCC 90,028, with curcumin prepared in 10% dimethyl sulfoxide (DMSO) and an LED device with 22.0 mW/cm² light intensity and 455 nm wavelength [3]. In other studies, curcumin demonstrated antifungal activity against clinical isolates of *C. albicans* and *C. parapsilosis*, with Minimum Inhibitory Concentrations (MICs) of 32 and 64 µg/mL, respectively [29]. Photoactivated curcumin with a 460 nm laser was active against *C. albicans* ATCC 10,231 and reduced viable yeast counts by 95% [7].

In our study, in addition to using different yeasts, we employed an LED device with lower light intensity (0.02165 W/cm²) and a wavelength of 450 nm. Therefore, the variability among these parameters may explain the differences observed between studies.

Photoactivated curcumin exerts its antifungal action predominantly through the generation of ROS, which induce generalized oxidative damage to cells and the extracellular matrix of the biofilm [12]. During early biofilm development, cells are more exposed and the extracellular matrix is still immature, which may facilitate photosensitizer access, oxygen diffusion, and ROS-mediated disruption of adhesion and microcolony formation. This may explain why curcumin-mediated aPDT was more effective in preventing biofilm formation than in disrupting mature biofilms [31]. Membrane destabilization by curcumin facilitates its internalization, intensifying the photodynamic effect. Intracellular ROS oxidize proteins, lipids, and nucleic acids, causing DNA damage and protein inactivation, leading to metabolic paralysis and cell death [12, 31].

Overall, these results indicate that curcumin-mediated aPDT is particularly effective as a preventive antibiofilm strategy, acting during the early stages of *Candida* biofilm development rather than primarily as a biofilm-eradicating intervention.

###  Effect of photodynamic therapy on mature biofilms

The efficacy of curcumin-mediated aPDT against mature yeast biofilms exhibited marked species-dependent variability (Table 2). The highest reduction in biofilm biomass was observed for *C. parapsilosis* ATCC 22,019 (49.63%), whereas *C. dubliniensis* ATCC MYA-646 showed minimal susceptibility, with only 0.64% inhibition. In the mature biofilm assay, data distribution was compatible with normality according to the Shapiro-Wilk test (*p* = 0.284). Nevertheless, due to the limited sample size and to preserve a conservative statistical framework comparable to that used for the biofilm formation assay, non-parametric analysis was maintained. The Kruskal–Wallis test showed no statistically significant differences between curcumin-mediated aPDT-treated mature biofilms and their corresponding controls (*p* > 0.05). Consequently, although reductions of up to 49.63% were observed, particularly for *C. parapsilosis* ATCC 22,019, these effects should be interpreted as species-dependent tendencies rather than statistically validated mature-biofilm disruption.

**Table 2:** OD_620nm_ of mature biofilms after curcumin-mediated phototherapy and non-photoactivated controls

Candida	Curcumin (320 µM)		Non- photoactivated controls		Reductions
	Mean	SD	Mean	SD	%
*C. albicans* ATCC 10,231	**0.270**	± 0.045	**0.332**	± 0.063	18.67
*C. metapsilosis* ATCC 96,143	**0.681**	± 0.147	**0.707**	± 0.103	3.68
*C. dubliniensis* ATCC MYA-646	**0.617**	± 0.105	**0.621**	± 0.167	0.64
*C. orthopsilosis* ATCC 96,141	**0.495**	± 0.093	**0.581**	± 0.133	14.80
*C. parapsilosis* ATCC 22,019	**0.275**	± 0.065	**0.546**	± 0.141	49.63
Non inoculated control	**0.058**	± 0.003	**0.057**	± 0.003	-

*SD* Standard Deviation, *ATCC* American Type Culture Collection

The low response observed for *C. dubliniensis* may be associated with intrinsic properties of mature biofilms, particularly the extracellular polymeric matrix, which limits photosensitizer penetration and ROS diffusion, as well as the presence of metabolically heterogeneous and persister cell populations, known to confer tolerance to oxidative stress [23]. In addition, species-specific differences in stress response mechanisms may further contribute to reduced susceptibility to aPDT [6].

These findings reinforce the role of biofilm maturity as a critical determinant of aPDT efficacy. Unlike early biofilms, mature biofilms possess a consolidated three-dimensional structure, increased extracellular matrix density, altered metabolic gradients, and subpopulations of cells with reduced susceptibility to oxidative damage.

Moreover, for mature biofilms, blue LED irradiation alone also did not substantially reduce established *Candida* biomass. The light-only controls showed OD620nm values of 0.332 ± 0.063 for *C. albicans*, 0.707 ± 0.103 for *C. metapsilosis*, 0.621 ± 0.167 for *C. dubliniensis*, 0.581 ± 0.133 for *C. orthopsilosis*, and 0.546 ± 0.141 for *C. parapsilosis*. Thus, although curcumin-mediated aPDT produced species-dependent reductions in mature biofilms, the light-only controls confirmed that irradiation alone was insufficient to disrupt established biofilm biomass.

Mature biofilms represent a significant therapeutic challenge, as their mature structure confers intrinsic resistance to antimicrobial agents through multiple mechanisms, such as physical barrier, metabolic heterogeneity, phenotypic alterations, and persistent cells [32].

Divergent results have been observed in previous studies evaluating the efficacy of antimicrobial photodynamic therapy against *Candida* biofilms compared to our findings. For instance, Ma et al., [17] irradiated *C. albicans* biofilms (CCA1, CCA2, and ATCC 90028) with LED (455 nm, 22.0 mW/cm²) for 6 min. This resulted in viability inhibition of 90.87% for ATCC 90,028, 66.44% for CCA1, and 86.74% for CCA2. Another in vivo study investigated the application of a curcumin solution (10 mL, 7.5 mg curcumin) sprayed into the oral cavity of oncology patients suffering from oral mucositis. After four weekly applications over one month, a reduction in colony-forming unit counts was observed in the later evaluations (21 and 30 days). Andrade et al., [4].

Although the results of previous studies evidenced that photoactivated curcumin was active against yeast biofilms, in our study the activity was more discrete, with inhibition values not exceeding 50% of the total biofilm biomass. Methodological differences between the studies, such as LED light intensity, experimental phototherapy evaluation models, and different strains analyzed, may have influenced the results.

Thus, the present findings reveal an important limitation of curcumin-mediated aPDT: while it strongly suppresses biofilm formation, its capacity to reduce pre-established mature biomass is more restricted and species dependent. This distinction is biologically relevant because mature biofilms are characterized by reduced photosensitizer penetration, limited oxygen availability in deeper layers, and increased tolerance to ROS-mediated injury [13, 16]. In addition to damaging fungal cells, reactive oxygen species (ROS) in biofilms also break down polysaccharides and other components of the extracellular matrix. This degradation compromises the biofilm’s three-dimensional structure and resistance [14]. The internalization of curcumin by cells in both growth forms, planktonic and biofilm, is documented and constitutes a determining factor for treatment efficiency, allowing the generation of ROS to occur in critical intracellular compartments [11, 26].

A thorough understanding of strategies to inhibit biofilm formation and to act against pre-formed biofilms is crucial for managing persistent infections and combating antimicrobial resistance [27, 30]. In this regard, aPDT offers a promising approach for combating *Candida* biofilm formation, especially in medical device-related infections.

The absence of relevant antibiofilm activity in the non-photoactivated controls supports the light-dependent nature of curcumin activity under the tested conditions. This finding indicates that photoactivation is required to enhance curcumin-mediated antibiofilm performance, consistent with the photochemical basis of aPDT. Mechanistically, this effect is interpreted in light of the well-established principles of aPDT. Upon irradiation, curcumin is excited from the ground state to an excited singlet state and subsequently undergoes intersystem crossing to a triplet state, as described by the Jablonski diagram [8]. This excited state enables interaction with O_2_ via Type I and Type II photochemical pathways, leading to the generation of ROS, which are widely recognized as the primary mediators of microbial inactivation in aPDT.

aPDT is a promising therapeutic strategy for combating microbial infections mediated by biofilms, particularly those caused by antimicrobial-resistant microorganisms. Given the current rise in infections that are resistant to conventional treatments, there is an increasing need for innovative approaches [19]. In this context, the present study supports curcumin-mediated aPDT primarily as a preventive antibiofilm strategy against *Candida* spp., while also indicating that optimization of irradiation parameters, photosensitizer delivery, and treatment protocols will be necessary to improve efficacy against mature biofilms.

##  Conclusions

This study examined the effectiveness of curcumin, when photoactivated by 450 nm LED light, against biofilms of five *Candida* species. We investigated its ability to both inhibit biofilm formation (preventive approach) and eradicate existing mature biofilms. Our findings indicate that photoactivated curcumin significantly inhibited biofilm development across all standard ATCC strains tested. Notably, *C. metapsilosis* and *C. parapsilosis* exhibited the highest susceptibility, with 86.72% and 83.26% inhibition, respectively. This inhibitory effect is likely associated with the reactive oxygen species (ROS) generated during photoactivation, which causes irreversible oxidative damage to vital cellular components, thereby impeding initial adhesion and the formation of the polymeric matrix.

While aPDT demonstrated species-dependent activity against 48-hour mature biofilms, its efficacy was significantly attenuated. *C. parapsilosis* showed the most substantial biomass reduction at 49.63%, whereas *C. dubliniensis* exhibited the lowest efficacy, with only a 0.64% reduction. This disparity suggests that the mature biofilm’s architecture acts as a physical barrier, impeding photosensitizer penetration and reactive oxygen species (ROS) diffusion. Furthermore, the presence of metabolically heterogeneous and persistent cell populations within the mature biofilm may contribute to its resistance. Curcumin-mediated aPDT showed a strong inhibitory effect on biofilm formation, indicating significant preventive potential. However, its efficacy against mature biofilms was limited and species-dependent, reflecting the intrinsic resistance of established biofilm structures. These findings indicate that aPDT is more effective as a preventive strategy rather than for the eradication of mature biofilms, without implying a direct evaluation of intermediate developmental stages. Therefore, optimizing photodynamic parameters and developing synergistic strategies to enhance photosensitizer penetration and overcome biofilm-associated resistance are essential to improve its therapeutic applicability.

## Data Availability

No datasets were generated or analyzed during the current study.

## References

[1] Ali A, Zahra A, Kamthan M, Husain FM, Albalawi T, Zubair M, Alatawy R, Abid M, Noorani MS (2023) Microbial biofilms: applications, clinical consequences, and alternative therapies. Microorganisms 11(8):1934. 10.3390/microorganisms11081934 37630494 10.3390/microorganisms11081934 PMC10459820

[2] Alnuaimi AD, O’Brien-Simpson NM, Reynolds EC, McCullough MJ (2013) Clinical isolates and laboratory reference *Candida* species and strains have varying abilities to form biofilms. FEMS Yeast Res 13(7):689–699. 10.1111/1567-1364.12068 23927631 10.1111/1567-1364.12068

[3] Andrade MC, Ribeiro APD, Dovigo LN, Brunetti IL, Giampaolo ET, Bagnato VS, Pavarina AC (2013) Effect of different pre-irradiation times on curcumin-mediated photodynamic therapy against planktonic cultures and biofilms of *Candida* spp. Arch Oral Biol 58(2):200–210. 10.1016/j.archoralbio.2012.10.011 23153629 10.1016/j.archoralbio.2012.10.011

[4] Andrade RCDV, Reis TA, Rosa LP, Santos GPO, Silva FC (2022) Comparative randomized trial study about the efficacy of photobiomodulation and curcumin antimicrobial photodynamic therapy as a coadjuvant treatment of oral mucositis in oncologic patients: antimicrobial, analgesic, and degree alteration effect. Support Care Cancer 30(9):7365–7371. 10.1007/s00520-022-07127-x 35608694 10.1007/s00520-022-07127-x

[5] Atriwal T, Azeem K, Husain FM, Hussain A, Khan MN, Alajmi MF, Abid M (2021) Mechanistic understanding of *Candida albicans* biofilm formation and approaches for its inhibition. Front Microbiol 12:638609. 10.3389/fmicb.2021.638609 33995297 10.3389/fmicb.2021.638609 PMC8121174

[6] Brown AJP, Budge S, Kaloriti D, Tillmann A, Jacobsen MD, Yin Z, Ene IV, Bohovych I, Sandai D, Kastora S, Potrykus J, Ballou ER, Childers DS, Shahana S, Leach MD (2014) Stress adaptation in a pathogenic fungus. J Exp Biol 217(1):144–155. 10.1242/jeb.088930 24353214 10.1242/jeb.088930 PMC3867497

[7] Daliri F, Azizi A, Goudarzi M, Lawaf S, Rahimi A (2019) In vitro comparison of the effect of photodynamic therapy with curcumin and methylene blue on *Candida albicans* colonies. Photodiagnosis Photodyn Ther 26:193–198. 10.1016/j.pdpdt.2019.03.017 30914389 10.1016/j.pdpdt.2019.03.017

[8] Dias LD, Blanco KC, Mfouo-Tynga IS, Inada NM, Bagnato VS (2020) Curcumin as a photosensitizer: from molecular structure to recent advances in antimicrobial photodynamic therapy. J Photochem Photobiol C Photochem Rev 45:100384. 10.1016/j.jphotochemrev.2020.100384

[9] Dovigo LN, Pavarina AC, Carmello JC, Machado AL, Brunetti IL, Bagnato VS (2011) Susceptibility of clinical isolates of *Candida* to photodynamic effects of curcumin. Lasers Surg Med 43(9):927–934. 10.1002/lsm.21110 22006736 10.1002/lsm.21110

[10] Dovigo LN, Pavarina AC, Ribeiro AP, Brunetti IL, Costa CADS, Jacomassi DP, Bagnato VS, Kurachi C (2011) Investigation of the photodynamic effects of curcumin against *Candida albicans*. Photochem Photobiol 87(4):895–903. 10.1111/j.1751-1097.2011b.00937.x 21517888 10.1111/j.1751-1097.2011.00937.x

[11] Du M, Su L, Zhu L, Li X, Li H, Ji H, Huang R, Zhou G (2021) Antimicrobial photodynamic therapy for oral *Candida* infection in adult AIDS patients: a pilot clinical trial. Photodiagnosis Photodyn Ther 34:102310. 10.1016/j.pdpdt.2021.102310 33901690 10.1016/j.pdpdt.2021.102310

[12] Dube E, Okuthe GE (2025) Nanocurcumin and curcumin-loaded nanoparticles in antimicrobial photodynamic therapy: Mechanisms and emerging applications. Micro MDPI. 10.3390/micro5030039

[13] Fan F, Liu Y, Liu Y, Lv R, Sun W, Ding W, Cai Y, Li W, Liu X, Qu W (2022) *Candida albicans* biofilms: antifungal resistance, immune evasion, and emerging therapeutic strategies. Int J Antimicrob Agents 60(5–6):106673. 10.1016/j.ijantimicag.2022.106673 36103915 10.1016/j.ijantimicag.2022.106673

[14] Garcia BA, de Lima JF, Urbano A, Alves F, Pelino JEP, de Andrade CR (2021) *Candida* biofilm matrix as a resistance mechanism against photodynamic therapy. Photodiagnosis Photodyn Ther 36:102525. 10.1016/j.pdpdt.2021.102525 34509685 10.1016/j.pdpdt.2021.102525

[15] Gourari-Bouzouina K, Bensouilah I, Sebane A, Ouchenane Z, Smati F (2024) Evaluation of mixed biofilm production by *Candida* spp. and *Staphylococcus aureus* strains co-isolated from cystic fibrosis patients in northwest Algeria. Diagn Microbiol Infect Dis 109(3):116321. 10.1016/j.diagmicrobio.2024.116321 38677054 10.1016/j.diagmicrobio.2024.116321

[16] Kaur J, Nobile CJ (2023) Antifungal drug-resistance mechanisms in *Candida* biofilms. Curr Opin Microbiol 71:102237. 10.1016/j.mib.2022.102237 36436326 10.1016/j.mib.2022.102237 PMC11569868

[17] Ma J, Shi H, Sun H, Li J, Bai Y (2019) Antifungal effect of photodynamic therapy mediated by curcumin on *Candida albicans* biofilms in vitro. Photodiagnosis Photodyn Ther 27:280–287. 10.1016/j.pdpdt.2019.06.015 31233886 10.1016/j.pdpdt.2019.06.015

[18] Melo AS, Bizerra FC, Freymüller E, Arthington-Skaggs BA, Colombo AL (2011) Biofilm production and evaluation of antifungal susceptibility amongst clinical *Candida* spp. isolates, including strains of the *Candida parapsilosis* complex. Med Mycol 49(3):253–262. 10.3109/13693786.2010.530032 21039308 10.3109/13693786.2010.530032

[19] Mishra R, Panda AK, De Mandal S, Shakeel M, Bisht SS, Khan J (2023) Therapeutic strategies against biofilm infections. Life (Basel) 13(1):172. 10.3390/life13010172 36676121 10.3390/life13010172 PMC9866932

[20] Peeters E, Nelis HJ, Coenye T (2008) Comparison of multiple methods for quantification of microbial biofilms grown in microtiter plates. J Microbiol Methods 72(2):157–165. 10.1016/j.mimet.2007.11.010 18155789 10.1016/j.mimet.2007.11.010

[21] Pereira R, Santos FRO, Brito EHS, Morais SM (2021) Biofilm of *Candida albicans*: formation, regulation and resistance. J Appl Microbiol 131(1):11–22. 10.1111/jam.14949 33249681 10.1111/jam.14949

[22] Ramage G, Borghi E, Rodrigues CF, Kean R, Williams C, Lopez-Ribot J (2023) Our current clinical understanding of *Candida* biofilms: where are we two decades on? APMIS 131(11):636–653. 10.1111/apm.13310 36932821 10.1111/apm.13310

[23] Ramage G, Rajendran R, Sherry L, Williams C (2012) Fungal biofilm resistance. Int J Microbiol 2012:528521. 10.1155/2012/528521 22518145 10.1155/2012/528521 PMC3299327

[24] Sanitá PV, Pavarina AC, Dovigo LN, Ribeiro APD, Andrade MC, Mima EGDO (2018) Curcumin-mediated anti-microbial photodynamic therapy against *Candida dubliniensis* biofilms. Lasers Med Sci 33:709–717. 10.1007/s10103-017-2382-8 29134404 10.1007/s10103-017-2382-8

[25] Sharma K, Parmanu PK, Sharma M (2024) Mechanisms of antifungal resistance and developments in alternative strategies to combat *Candida albicans* infection. Arch Microbiol 206(3):95. 10.1007/s00203-023-03824-1 38349529 10.1007/s00203-023-03824-1

[26] Songca SP, Adjei Y (2022) Applications of antimicrobial photodynamic therapy against bacterial biofilms. Int J Mol Sci 23(6):3209. 10.3390/ijms23063209 35328629 10.3390/ijms23063209 PMC8953781

[27] Su Y, Yrastorza JT, Matis M, Cusick J, Zhao S, Wang G, Xie J (2022) Biofilms: formation, research models, potential targets, and methods for prevention and treatment. Adv Sci 9(29):e2203291. 10.1002/advs.202203291 10.1002/advs.202203291 PMC9561771 36031384

[28] Thomaz DY, Almeida Jr JN, Lima GME, Nunes MDO, Camargo CH, Grenfell RDC, Benard G, Del Negro GM (2018) An azole-resistant *Candida parapsilosis* outbreak: Clonal persistence in the intensive care unit of a Brazilian teaching hospital. Front Microbiol 9:2997. 10.3389/fmicb.2018.02997 30568646 10.3389/fmicb.2018.02997 PMC6290035

[29] Tsao SM, Yin MC (2000) Enhanced inhibitory effect from interaction of curcumin with amphotericin B or fluconazole against *Candida* species. J Food Drug Anal 8(3):208–212. 10.38212/2224-6614.2831

[30] Upadhyay A, Jaiswal N, Kumar A (2025) Biofilm battle: New transformative tactics to tackle the bacterial biofilm infections. Microb Pathog 199:107277. 10.1016/j.micpath.2025.107277 39756524 10.1016/j.micpath.2025.107277

[31] Warrier A, Mazumder N, Prabhu S, Satyamoorthy K, Murali TS (2021) Photodynamic therapy to control microbial biofilms. Photodiagn Photodyn Ther 33:102090. 10.1016/j.pdpdt.2020.102090 10.1016/j.pdpdt.2020.102090 33157331

[32] Xu Z, Liang Y, Lin S, Chen D, Li B, Li L, Deng Y (2022) Regulatory network controls microbial biofilm development, with *Candida albicans* as a representative: From adhesion to dispersal. Bioengineered 13(1):253–267. 10.1080/21655979.2021.1996747 34709974 10.1080/21655979.2021.1996747 PMC8805954

[33] Zayed SM, El-Sayed HS, Ghazy AA (2021) Biofilm formation by *Streptococcus mutans* and its inhibition by green tea extracts. AMB Express 11(1):73. 10.1186/s13568-021-01232-6 34032940 10.1186/s13568-021-01232-6 PMC8149520

